# Gradient Joule Heating Curing Performance of Steel-Fiber-Reinforced High-Performance Concrete in Severe Cold Environments: A Preliminary Attempt for Deep-Cold Concrete Construction

**DOI:** 10.3390/ma18122909

**Published:** 2025-06-19

**Authors:** Xinyu Liu, Jinghui Wang, Zheng Zhou, Lei Zhang, Qiang Fu

**Affiliations:** 1 The School of Art and Design, Zhejiang Business College, Hangzhou 310053, China; 2 The School of Construction and Engineering, Zhejiang College of Construction, Hangzhou 311231, China; 3 School of Civil Engineering, Harbin Institute of Technology, Harbin 150090, China; 4 School of Water Conservancy and Civil Engineering, Northeast Agricultural University, Harbin 150030, China

**Keywords:** gradient Joule heating curing, winter concrete construction, mechanical properties, microstructural analysis

## Abstract

Winter concrete construction in cold regions faces significant challenges due to extreme subzero temperatures, and the harsh environment presents new requirement for cement-based materials to resist this hostile external condition. To address this gap, this study proposes gradient Joule heating (GJH) curing for steel-fiber-reinforced high-performance concrete (SFR-HPC) in subzero environments (−20 °C to −60 °C). Compared to room-temperature (RT) curing, GJH enabled specimens at −20 °C to −50 °C to achieve equivalent mechanical properties within a short curing duration; the compressive strength of the specimens cured at such low environmental temperature still reached up to that of the specimen cured by RT curing. Moreover, the compressive strength of the specimens cured at −60 °C retained >60 MPa despite reduced performance. Specifically, the specimens cured at −20 °C, −30 °C, −40 °C, and −50 °C for 2 days exhibited compressive strengths of 75.8 MPa, 79.2 MPa, 77.6 MPa, and 75.4 MPa, respectively. FTIR/XRD confirmed that the specimens cured by GJH showed hydration product integrity akin to RT-cured specimens. Moreover, it should be noted that early pore structure deteriorated with decreasing temperatures, but prolonged curing mitigated these differences. These results validate GJH as a viable method for in situ HPC production in extreme cold, addressing critical limitations of conventional winter construction techniques.

## 1. Introduction

High-performance concrete (HPC) as an essential cement-based engineering material has gained widespread application in construction due to its exceptional mechanical properties (typically with compressive strength exceeding 60 MPa), remarkable toughness, and outstanding durability [1,2]. Compared with conventional concrete, HPC achieves significantly enhanced structural performance through optimized mix design featuring reduced water-to-binder ratios, finer aggregate gradation, and incorporation of reinforcing materials such as steel fibers or carbon fibers [3,4]. The complex winter climate conditions in cold regions pose substantial challenges to normal concrete construction, severely impeding local infrastructure development and adversely affecting regional economic growth [5,6]. Given the strict performance requirements imposed by these extreme environmental conditions, the successful implementation of HPC in polar and cold-region engineering projects holds significant practical importance for advancing development strategies in harsh environments [7,8]. The development of HPC under subzero temperatures has emerged as a critical scientific challenge in polar and cold regions characterized by prolonged winters and ultra-low temperatures [9]. Conventional winter construction techniques, including thermal insulation, external heating, material preheating, and chemical admixture methods, exhibit significant limitations in practical applications. To be specific, these techniques either require external facilities to achieve curing of fresh cement-based materials under subzero temperatures or exhibit insufficient effectiveness in stimulating strength development under such conditions. In conclusion, these methods demonstrate inconsistent effectiveness and restricted applicability and lack quantitative evaluation criteria, failing to meet the stringent quality requirements for HPC in subzero environments [10,11,12,13]. Consequently, developing an innovative curing method for in situ preparation of HPC under subzero environments has become imperative.

Joule heating (JH) curing technology, as an innovative concrete curing method, has attracted increasing research attention for its ability to provide controlled curing temperatures through electrical resistance heating [14,15]. However, current research presents notable deficiencies: inconsistent curing durations and imprecise temperature control compromise performance reproducibility [16,17]. Furthermore, existing studies predominantly focused the development of electrothermal functionalities of the composites (e.g., deicing or structural health monitoring) [18,19], while overlooking its potential in curing applications [20,21]. Although some studies focused on the winter concrete construction issue, the environmental temperature was limited above −20 °C, which is mild compared to the harsh environment in polar regions [22,23]. The distinctive heating mechanism of JH curing demonstrates considerable promise for addressing HPC challenges in subzero environments. Nevertheless, under harsh environments below −20 °C, rapid temperature rise during JH curing creates substantial thermal gradients that may induce early-age cracking and compromise long-term performance [24]. Therefore, establishing optimized JH curing regimes for extreme cold conditions represents an urgent technological challenge.

The gradient curing strategy is expected to address the aforementioned issues. In the first stage, the temperature of the specimen is maintained at approximately room temperature, which is conducive to the development of early-stage strength, thereby enabling the specimen to withstand potential thermal expansion effects caused by temperature differences in later stages. Subsequently, the curing temperature in the next stage is kept within a range that promotes the hydration reaction of HPC, thereby facilitating the strength development of the specimen.

This work innovatively employed a gradient Joule heating (GJH) curing method to fabricate steel-fiber-reinforced high-performance concrete (SFR-HPC) specimens under subzero temperatures ranging from −20 °C to −60 °C. Comparative analysis of early-age and 28-day performance evolution elucidated the influence of GJH curing on mechanical properties and microstructural development in HPC. The technical feasibility of this method for HPC production under extreme cold conditions was systematically evaluated, with particular emphasis on long-term strength development characteristics of SFR-HPC. Microstructural characterization techniques including Fourier-transform infrared spectroscopy (FTIR), X-ray diffraction (XRD) and mercury intrusion porosimetry (MIP) were employed to investigate the correlation between macroscopic properties and microstructural evolution under varying curing regimes, durations, and temperature conditions. These findings provided both theoretical foundation and practical guidance for advancing HPC construction technologies in subzero environments.

## 2. Materials and Methods

### 2.1. Raw Materials

The experiment utilized P.O 42.5 cement produced by Harbin Yatai Cement Plant (Changchun, China). The silica fume was supplied by Elkem Company (Oslo, Norway) to serve as the supplementary cementitious material, and the average particle size of the silica fume was 0.18 μm, with a density of 2.2 g/cm^3^. The chemical compositions of the cement and silica fume are presented in Table 1. For the preparation of steel-fiber-reinforced high-performance concrete (SFR-HPC), quartz sand with a particle size range of 0.84–2 mm was selected as fine aggregate, showing a specific gravity of 2.65. Additionally, the steel fibers used in the experiment had a diameter of 0.22 mm and a length of 13 mm. To ensure adequate fluidity of the fresh SFR-HPC, a polycarboxylate ether-based superplasticizer (PCE-SP) produced by Jiangsu Sobute New Materials Co., Ltd. (Nanjing, China), was incorporated.

### 2.2. Mixing Proportion and Preparation of SFR-HPC Specimens

The mixed proportions of raw materials for the SFR-HPC are illustrated in Table 2. The cementitious materials consist of cement and silica fume, with the PCE-SP dosage being 2.5% by mass of the cementitious materials. The preparation procedure of SFR-HPC is detailed as follows: Cement and silica fume were first dry-mixed in the mixing container at high speed for 3 min. Then, the mixed solution containing water and PCE-SP was poured into the mixer. The mixture was mixed at low speed for 2 min followed by high-speed mixing for 3 min. Subsequently, quartz sand was added and mixed at high speed for 5 min. Afterward, all steel fibers were gradually added into the mixture while maintaining slow mixing for 5 min to achieve uniform dispersion within the fresh paste. This was followed by additional high-speed mixing for 8 min to further enhance the homogeneity of steel fiber distribution in the SFR-HPC matrix. Finally, the fresh mixture was cast into plastic molds measuring 40 mm × 40 mm × 160 mm^3^, which were then covered with plastic wrap to prevent moisture evaporation.

### 2.3. Curing Regime

**Figure 1:**
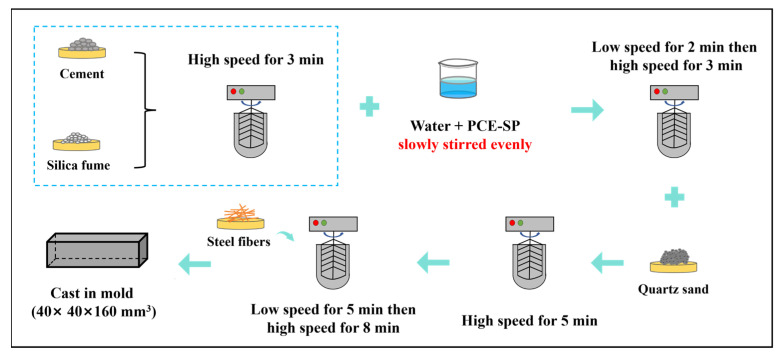
Schematic diagram of preparation method.

In this work, the SFR-HPC specimens were cured over 3 d, with 28 d room-temperature (RT) curing (20 ± 2 °C, RH > 95%) as the comparison. Moreover, the SFR-HPC specimens were subjected to a 12 h GJH curing process in different negative-temperature environments, and a gradient curing regime was conducted. As illustrated in Figure 1, this curing regime consisted of two distinct stages: During the initial phase (0–6 h), the specimens were maintained at 25 ± 2 °C to ensure the development of sufficient early-age structural strength, mitigating potential thermal stress induced by subsequent high-temperature curing [25,26]. In the second stage (6–12 h), the curing temperature was elevated to 60 ± 2 °C to accelerate the hydration reaction, to promote strength development [27].

To be more precise, a dynamic power regulation strategy was implemented during the curing process, wherein the applied electrical power was automatically adjusted based on real-time temperature monitoring. Specifically, the power input was increased when the measured curing temperature fell below the target value and decreased when it exceeded the setpoint, ensuring precise temperature control. Following the 12 h GJH curing, all specimens were transferred to the RT curing environment for further hydration. To systematically evaluate the effectiveness of this curing method, various tests and analyses were conducted on the GJH-cured specimens at curing ages of 2 and 28 days. Comparative analyses were performed with 3-day and 28-day RT cured specimens, respectively, aiming to elucidate the influence of GJH curing on both early-age and long-term mechanical properties of SFR-HPC.

### 2.4. Temperature Monitoring

The temperatures of SRF-HPC specimens during GJH curing were monitored using a multi-channel temperature acquisition system. A thermocouple was embedded at the center of each specimen to record the internal temperature. To prevent potential measurement interference caused by current flow through the thermocouple during GJH curing, the thermocouple was insulated using adhesive tape. The temperature acquisition system was configured to record the central temperature of the specimens at 30 s intervals throughout the GJH curing process.

### 2.5. Mechanical Property Test

SFR-HPC specimens were prepared with sizes of 40 mm × 40 mm × 160 mm for mechanical property testing. For the compressive strength tests, a constant loading rate of 2400 ± 200 N/s was applied, and six specimens were tested, with the average value taken as the final result. The flexural strength was determined using a three-point bending test method. During testing, the loading rate was strictly controlled at 50 ± 10 N/s. To be more specific, six specimens were prepared for the strength test, and the average was taken as the result.

### 2.6. Microstructure Characterization

This work systematically investigated the performance evolution of SFR-HPC under different curing conditions, ages, and environment temperatures using multiple microstructural characterization techniques. For sample preparation, the specimens reaching the designated ages were first subjected to mechanical property testing and then crushed into small pieces. The hydration reaction was terminated by immersing the fragments in absolute ethanol for 48 h, followed by drying in a constant-temperature oven at 60 °C for another 48 h. The dried samples were ground and sieved through a 200-mesh sieve to prepare powder samples for Fourier transform infrared spectroscopy (FTIR) and X-ray diffraction (XRD) analyses. For mercury intrusion porosimetry (MIP), the hydrated and dried small pieces were used. To be specific, FTIR spectroscopy was performed using a Nicolet IS50 spectrometer (Thermo Fisher Scientific, Waltham, MA, USA). XRD analysis was carried out on an X’Pert Pro diffractometer (PANalytical, Almelo, The Netherlands) equipped with an X’Celerator detector. A Cu Kα radiation source (λ = 1.54 Å) was used at 45 kV and 40 mA. The measurements were conducted in Bragg–Brentano geometry with a scanning range of 5–45° (2θ), a step size of 0.033°, and a scanning time of approximately 10 min per sample. MIP analysis was conducted using an AutoPore IV 9510 porosimeter (Micromeritics, Norcross, GA, USA) with an applied pressure range of 0–60,000 psi.

## 3. Results and Discussion

### 3.1. The Temperature Development Regulation of GJH-Cured Specimens

**Figure 2:**
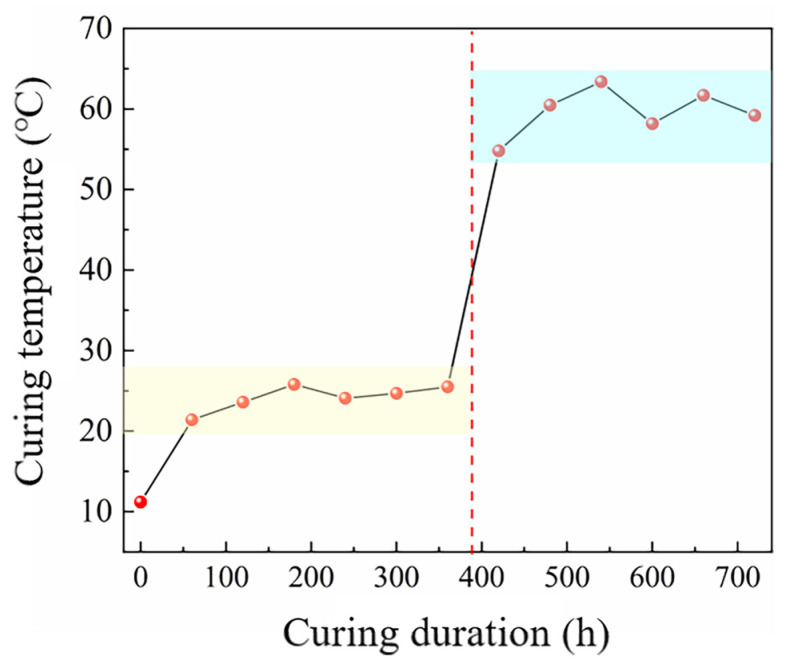
Experimental temperature development of GJH curing.

**Figure 3:**
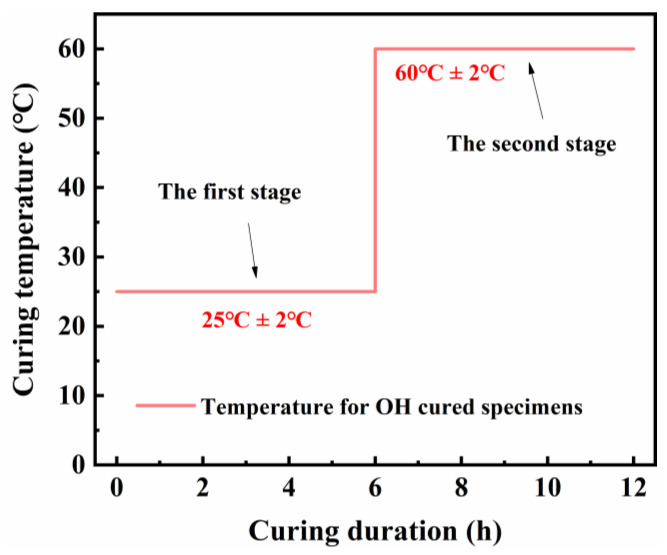
Diagram of procedure for gradient Joule heating curing.

Figure 2 depicts the temperature evolution curve of SFR-HPC specimens during GJH curing, recorded under an environmental temperature of −40 °C. As illustrated in Figure 2, the actual temperature development of the specimens exhibited excellent agreement with the schematic temperature profile shown in Figure 3. Specifically, during the first stage, the curing temperature remained consistently stable between 20 °C and 25 °C. Following an adjustment of the electrical power input at the sixth hour of GJH curing, the temperature of the specimens stabilized within the range of 55 °C to 60 °C, an optimal temperature range for promoting hydration of SFR-HPC under subzero temperature conditions. These observations demonstrate both the flexibility and controllability of the GJH curing method, further highlighting its advantages for concrete construction in cold regions during winter seasons with subzero temperatures.

### 3.2. Mechanical Properties of SFR-HPC Specimens Cured by GJH Curing

#### 3.2.1. Early-Age Mechanical Properties

**Figure 4:**
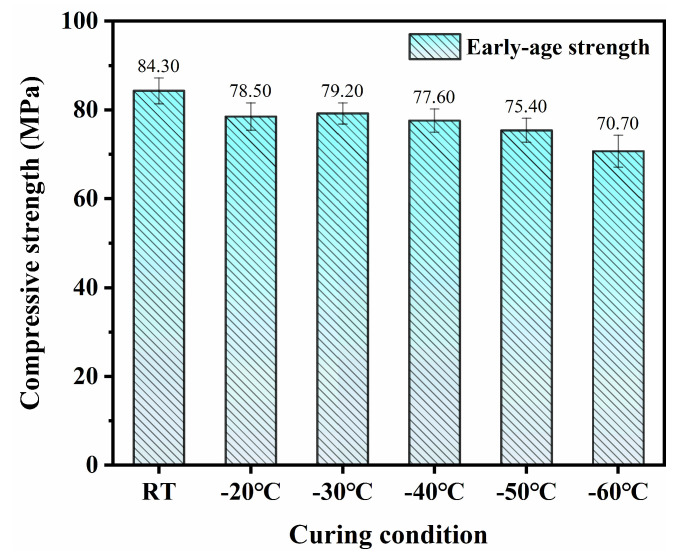
Early-age compressive strength of SFR-HPC specimens.

**Figure 5:**
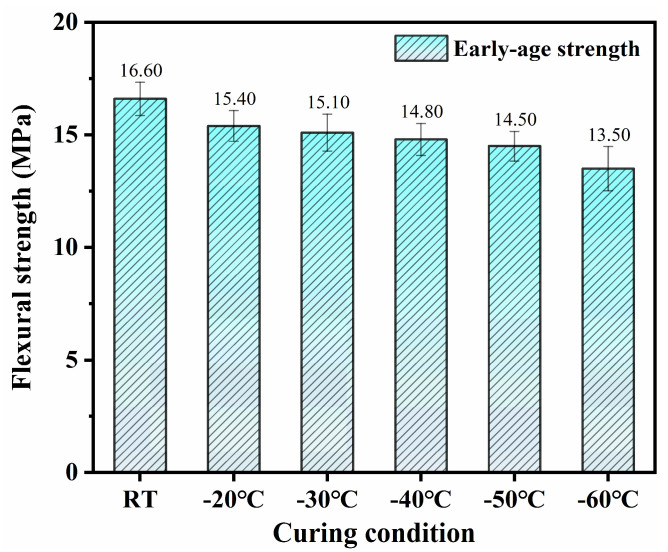
Early-age flexural strength of SFR-HPC specimens.

The development of early-age mechanical properties in SFR-HPC specimens subjected to GJH curing under different environmental temperatures is illustrated in Figure 4 and Figure 5. For reference, the mechanical properties of specimens cured by 3-day RT curing are also presented. The experimental results demonstrated that SFR-HPC exhibited relatively stable mechanical properties within the subzero temperature range of −20 °C to −60 °C, as exhibited in Figure 4. To be specific, the 3-day RT-cured specimens achieved the highest compressive strength of 84.3 MPa. In contrast, the compressive strengths of GJH-cured specimens under different subzero temperatures were as follows: −20 °C (78.5 MPa), −30 °C (79.2 MPa), −40 °C (77.6 MPa), −50 °C (75.4 MPa), and −60 °C (70.7 MPa). Compared to the reference group, the corresponding strength reductions were 6.9%, 6%, 7.9%, 10.6%, and 16.1%, respectively. Notably, except for the extreme condition of −60 °C, the differences in compressive strength among the specimens cured at other subzero temperatures remained within an acceptable margin of error. Comprehensive analysis revealed that when the environment temperature was no lower than −50 °C, specimens subjected to 12 h of GJH curing followed by 36 h of RT curing maintained compressive strengths above 75 MPa, demonstrating comparable mechanical properties to the 3-day RT-cured specimens. Even under the extreme condition of −60 °C, the specimens still attained a compressive strength of 70.7 MPa. These findings confirm that GJH curing significantly enhances the early-age mechanical properties of HPC in ultra-low-temperature environments.

It can be seen from Figure 5 that the flexural strengths of SFR-HPC specimens cured by GJH exhibited a similar development trend to their compressive strength. The 3-day RT-cured specimens achieved a flexural strength of 16.6 MPa, while those subjected to GJH curing at subzero temperatures of −20 °C, −30 °C, −40 °C, −50 °C, and −60 °C attained flexural strengths of 15.4 MPa, 15.1 MPa, 14.8 MPa, 14.5 MPa, and 13.5 MPa, respectively. This investigation demonstrates that the GJH curing method effectively facilitates significant strength development in SFR-HPC under subzero temperature conditions. We should note that, even at the ultra-low temperature of −60 °C, the specimens maintained a considerable flexural strength of 13.5 MPa. These findings not only address the long-standing challenge of concrete strength development in subzero environments but also provide a reliable technical solution for the construction of HPC structures in cold regions, highlighting its substantial engineering significance.

#### 3.2.2. Long-Term Mechanical Properties

**Figure 6:**
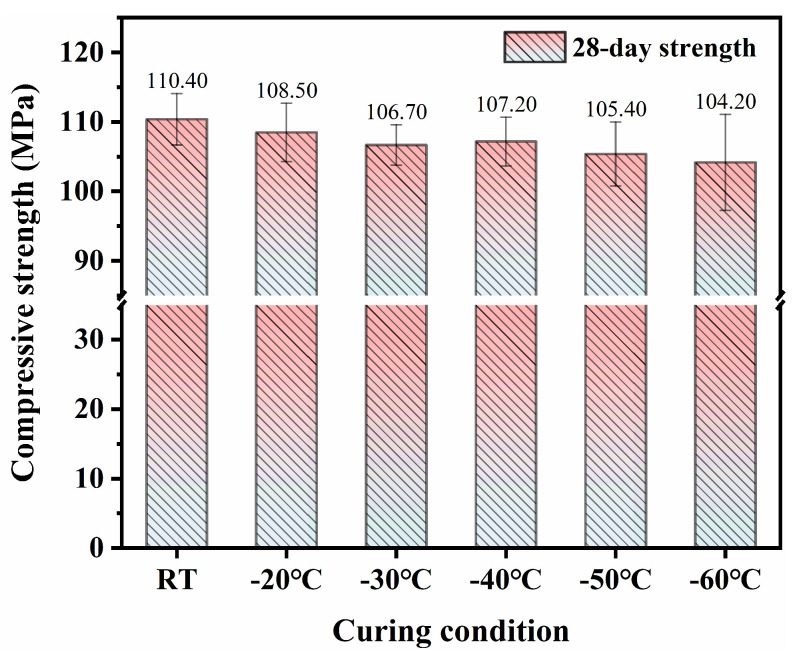
The 28-day compressive strength of SFR-HPC specimens.

**Figure 7:**
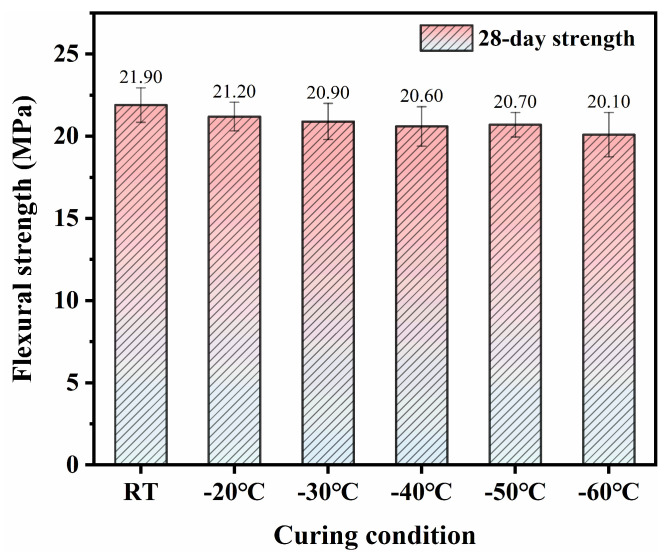
The 28-day flexural strength of SFR-HPC specimens.

Figure 6 and Figure 7 present the long-term mechanical properties of SFR-HPC specimens cured by GJH curing at different environmental temperatures. The results demonstrated that the long-term mechanical property development was not significantly affected by the early-age GJH curing. Specifically, the 28-day RT-cured SFR-HPC specimens achieved a compressive strength of 110.4 MPa, while those subjected to 12 h GJH curing at −20 °C, −30 °C, −40 °C, −50 °C, and −60 °C exhibited 28-day compressive strengths of 108.5 MPa, 106.7 MPa, 107.2 MPa, 105.4 MPa, and 104.2 MPa, respectively. These results indicated that all GJH-cured specimens under various subzero temperatures showed favorable strength development trends, maintaining comparable compressive strength levels to the RT-cured specimens. A similar development tendency was observed for flexural strength, with the 28-day RT-cured specimens reaching 21.9 MPa, and the GJH-cured specimens reached 21.1 MPa (−20 °C), 20.9 MPa (−30 °C), 20.6 MPa (−40 °C), 20.7 MPa (−50 °C), and 20.1 MPa (−60 °C), demonstrating minimal variations in flexural strength among different curing conditions.

The comprehensive long-term mechanical property results confirm that GJH curing, as an innovative concrete curing method, not only significantly enhances the early-age mechanical properties of SFR-HPC in subzero temperatures but also ensures favorable long-term performance development. The limited influence of initial subzero curing temperatures on the mechanical properties suggests that the GJH curing method holds great potential for preparing HPC in extremely cold environments.

#### 3.2.3. The Ratio of Flexural to Compressive Strength

**Figure 8:**
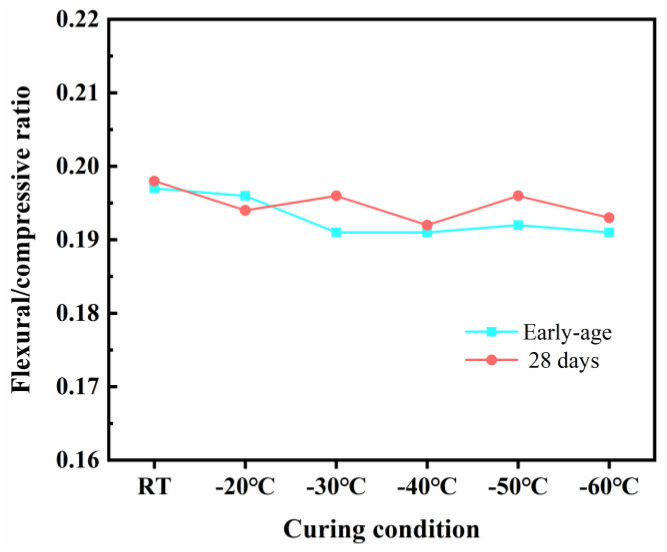
Ratios between flexural and compressive strengths of SFR-HPC specimens with different curing conditions.

The ratios of flexural to compressive strength at early age and 28 days were analyzed, as depicted in Figure 8. The results demonstrated that the ratios of flexural to compressive strength of SFR-HPC specimens under various curing conditions remained remarkably consistent across different curing ages. This observation indicated that the strength development regulations were essentially similar among all curing conditions as the hydration process progressed, suggesting that different initial curing temperatures did not adversely affect the long-term performance evolution of SFR-HPC. The strength growth characteristics aligned well with typical HPC behavior, further validating the reliability of GJH curing as an innovative curing method for HPC in different subzero temperature environments.

Based on the above results, the influence of GJH curing on the macroscopic property development of SFR-HPC under various subzero temperatures was explicated. To gain deeper insights into microstructural evolution mechanisms, comprehensive characterization techniques including XRD, FTIR and MIP were employed to examine the SFR-HPC specimens. For comparative analysis, specimens cured by RT curing along with those subjected to GJH curing at −20 °C, −40 °C, and −60 °C were specifically selected.

### 3.3. Hydration Product Analysis

#### 3.3.1. Fourier Transform Infrared (FT-IR) Spectroscopy

**Figure 9:**
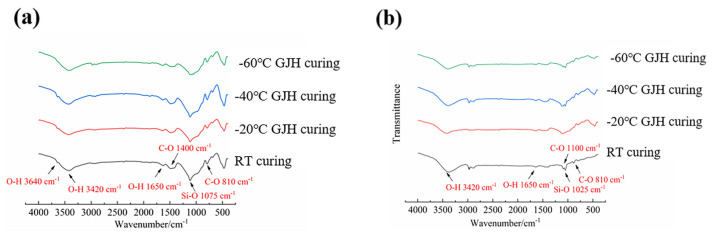
FTIR spectra of specimens subjected to different curing conditions: (**a**) at early age and (**b**) 28 days.

Figure 9a exhibits the FTIR spectra of specimens subjected to 2-day curing, and Figure 9b displays the FTIR spectra of specimens subjected to 28-day curing. It can be illustrated that the broad peak at 3420 cm^−1^ corresponds to the O–H stretching vibration of water molecules, while the peak at 3640 cm^−1^ originates from the O–H vibration in the hydration product Ca(OH)_2_. The weak absorption band near 1640 cm^−1^ was attributed to the bending vibration of O–H in water molecules [28,29]. The absorption band at 1400 cm^−1^ was associated with the presence of calcite, and the characteristic peak at 1075 cm^−1^ arose from the Si–O vibration in C–S–H gel. Meanwhile, the peak near 810 cm^−1^ was related to the vibrational mode of CO_3_^2−^. The FTIR results demonstrated that the characteristic peaks of early hydration products in SFR-HPC remain consistent across different curing conditions, indicating similar functional group compositions. This finding confirms that GJH curing, as a novel curing method, does not induce anomalous physicochemical reactions or generate additional hydration products in SFR-HPC, even under ultra-low temperature (−60 °C). The study provides critical theoretical support for the feasibility of using GJH curing to promote early-age strength development in SFR-HPC under ultra-low-temperature environments.

Figure 9b illustrates the FTIR spectra of SFR-HPC after 28 days of curing. Comparative spectral analysis revealed the complete disappearance of the characteristic peak at ~3640 cm^−1^, which was previously assigned to the O-H stretching vibration of Ca(OH)_2_ in Figure 9a. This significant spectral change indicated the near depletion of Ca(OH)_2_ content, demonstrating that the pozzolanic reaction between silica fume and Ca(OH)_2_ was effectively completed during the subsequent standard curing process. These results confirm that SFR-HPC possesses excellent hydration reactivity even after GJH curing, which contributes to the development of favorable mechanical properties. Regarding the hydration product systems, the 28-day-cured specimens exhibited identical spectral characteristics to their early-age counterparts. The hydration product systems remain consistent across different curing conditions, further verifying the stability of the material’s hydration process under varying curing conditions.

#### 3.3.2. Phase Composition

**Figure 10:**
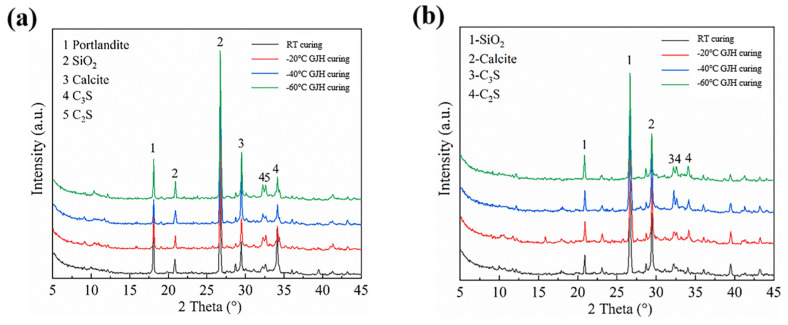
XRD patterns of specimens subjected to different curing conditions: (**a**) at early age and (**b**) 28 days.

The XRD analysis results of SFR-HPC specimens under different curing conditions are exhibited in Figure 10. The XRD patterns of early-age (2-day) specimens revealed that the primary crystalline phases in both RT-cured and GJH-cured SFR-HPC include Ca(OH)_2_, SiO_2_, calcite, C_3_S, and C_2_S. The identical phase composition observed under different curing methods indicated that the hydration product systems in SFR-HPC remain consistent between GJH curing and RT curing conditions, which corroborates the FTIR analysis findings. For 28-day-cured specimens, the XRD patterns demonstrated that while the hydration product systems maintain consistency across different curing methods, the predominant phases evolve to SiO_2_, calcite, C_3_S, and C_2_S, with the notable absence of Ca(OH)_2_ diffraction peaks. This observation confirmed the consumption of Ca(OH)_2_ by pozzolanic reaction during prolonged hydration, a phenomenon that aligns well with the FTIR analysis results. Comparative analysis of XRD patterns between different curing ages clearly showed significantly reduced characteristic peak intensities of cementitious components (C_3_S and C_2_S) in 28-day specimens compared to their early-age counterparts. This reduction demonstrated a more complete hydration reaction with extended curing duration, leading to substantial consumption of cement clinker constituents. These microstructural developments directly contribute to the remarkable enhancement in mechanical properties observed in SFR-HPC specimens.

### 3.4. Pore Structure Analysis

#### 3.4.1. The Pore Distribution of the Specimens at Early Age

**Figure 11:**
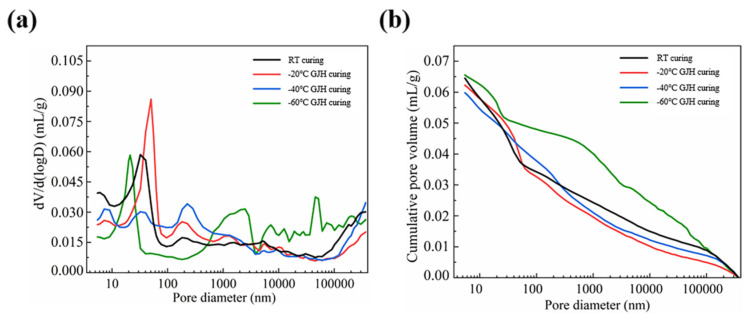
Pore structure of the specimens subjected to different curing conditions at early age: (**a**) pore size distribution and (**b**) cumulative pore volume.

The early-age pore size distribution and cumulative pore distribution of SFR-HPC under different curing conditions are depicted in Figure 11, with detailed pore structure parameters provided in Table 3. As observed in Figure 11, the RT-cured specimens exhibited a most probable pore size at approximately 32 nm. For the specimens cured under GJH curing at −20 °C, the most probable pore size slightly increased to around 50 nm. The −40 °C GJH-cured specimens displayed a bimodal distribution, with the most probable pore sizes located at approximately 32 nm and 226 nm. In contrast, the −60 °C GJH-cured specimens showed a more complex multimodal distribution, with the most probable pore sizes appearing at around 21 nm, 2488 nm, and 5440 nm. Notably, as the curing temperature decreased, the pore size distribution exhibited a clear coarsening trend. Specifically, the most probable pore size of the −40 °C GJH-cured specimens reached to 226 nm, falling into the harmful pore range, and the −60 °C GJH-cured specimens exceeded 1000 nm. Such significant pore coarsening inevitably adversely affects the mechanical properties of HPC. This finding aligns with the early-age mechanical property test results, where the −60 °C GJH-cured specimens demonstrated significantly lower mechanical strength than those under other curing conditions. The primary reason may be attributed to the substantial temperature difference (up to 120 °C) between the specimens and the environment temperature under −60 °C curing, leading to pronounced thermal stress during early-age curing and subsequent pore structure deterioration.

Table 3 provides detailed pore structure parameters for specimens under different curing conditions. Based on the influence of pore size on mechanical properties of HPC, the pores were classified into four categories: harmless pores (<20 nm), less harmful pores (20–100 nm), harmful pores (100–1000 nm), and severely harmful pores (>1000 nm) [30]. The analysis revealed that the RT-cured specimens had the highest proportion of harmless pores, while the GJH-cured specimens at different temperatures showed comparable harmless pore ratios. Notably, the −20 °C GJH-cured specimens exhibited the most favorable pore structure characteristics, with the proportions of less harmful pores (20–100 nm) and harmful pores (100–1000 nm) increasing by 5.1% and 6.6%, respectively, compared to the RT-cured specimens. This suggests that moderate subzero-temperature GJH curing helped refine the pore structure and enhance matrix densification. When the curing temperature decreased to −40 °C, the optimization effect of GJH curing was weakened, as the proportion of pores below 100 nm was lower than that of the RT-cured specimens. However, the proportion of severely harmful pores (>1000 nm) was significantly reduced, indicating this curing method still contributed to pore structure refinement. However, when the curing temperature further decreased to −60 °C, the pore structure exhibited significant deterioration: the proportions of pores in the <20 nm, 20–100 nm, and 100–1000 nm ranges decreased by 6.1%, 15.2%, and 2%, respectively, and the proportion of severely harmful pores (>1000 nm) sharply increased to 60.7%. This substantial degradation in pore structure is primarily attributed to the microstructural damage induced by the extreme temperature gradient under ultra-low-temperature curing conditions.

#### 3.4.2. The Pore Distribution of the Specimens at 28 Days

**Figure 12:**
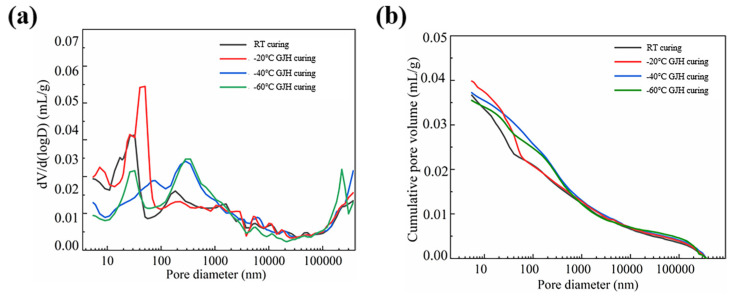
Pore structure of the specimens subjected to different curing conditions at 28 days: (**a**) pore size distribution and (**b**) cumulative pore volume.

The pore size distribution and cumulative pore volume of SFR-HPC specimens under different initial curing conditions after 28 days of continuous curing are presented in Figure 12. Compared to the early-age specimens, the pore structure distribution of the 28-day specimens exhibited more pronounced regularity. Specifically, the RT-cured specimens showed a single most probable pore size at ~26 nm; the −20 °C GJH-cured specimens exhibited its most probable pore size at ~50.36 nm. For the −40 °C GJH-cured specimens, the most probable pore size increased to ~283 nm. In contrast, the −60 °C GJH-cured specimens displayed a bimodal distribution, with the two most probable pore sizes at ~32 nm and ~226 nm. The results indicated that as the curing age increased, the most probable pore sizes of all specimens demonstrated a clear decreasing trend, confirming the significant improvement in pore structure due to prolonged curing. Although GJH curing under ultra-low temperatures had a certain adverse effect on the pore structure of concrete, this influence was reversible and did not fundamentally damage the material’s microstructure. Long-term observation revealed that with continued curing, the pore structure of the specimens was progressively optimized, and the performance differences among specimens subjected to different curing conditions gradually diminished, demonstrating favorable long-term development characteristics.

## 4. Conclusions

In this work, GJH curing was investigated as a novel method for SFR-HPC under subzero temperatures (−20 °C, −30 °C, −40 °C, −50 °C, and −60 °C). Its effects on the mechanical properties and microstructure of SFR-HPC were systematically compared with RT curing. The preliminary conclusions are as follows:

(1) GJH curing can effectively promise the mechanical strength development of SFR-HPC in subzero environments while ensuring favorable long-term performance development. The specimens cured at −20 °C, −30 °C, −40 °C, and −50 °C for 2 days exhibited compressive strengths of 75.8 MPa, 79.2 MPa, 77.6 MPa, and 75.4 MPa, respectively, and flexural strengths of 15.4 MPa, 15.1 MPa, 14.8 MPa, and 14.5 MPa, respectively. These results were comparable to those subjected to RT curing for 3 days (compressive strength: 84.3 MPa; flexural strength: 16.6 MPa). At −60 °C, the mechanical properties exhibited a declining trend but still exceeded 60 MPa and 13 GPa, confirming the feasibility and effectiveness of GJH curing.
(2) GJH curing does not adversely affect the formation of hydration products in SFR-HPC. FTIR and XRD analyses confirmed that the hydration products under GJH curing remained consistent with those under RT curing across all temperatures, demonstrating the stability of GJH curing.
(3) As the environment temperature decreased, the pore structure of SFR-HPC cured by GJH curing showed obvious deterioration at early age, while specimens at −20 °C exhibited a more refined pore structure than RT-cured specimens, and those at −40 °C and −60 °C displayed inferior porosity. To be specific, the pore size distributions (>1000 nm) of the cured specimens under RT curing, −20 °C, −40 °C, and −60 °C were 37.4%, 32.0%, 36.8%, and 60.7%, respectively. However, the differences in pore structure diminished with prolonged curing duration; the most probable pore size of the specimens cured at −60 °C for 28 days decreased to ~226 nm.

## Figures and Tables

**Table 1:** The chemical compositions of cement and silica fume.

Materials	SiO_2_	Al_2_O_3_	Fe_2_O_3_	MgO	CaO	SO_3_
Cement	20.86	5.47	3.94	1.73	62.23	2.66
Silica fume	95.8	0.8	0.2	0.2	0.5	-

**Table 2:** Mix proportion for SFR-HPC specimens.

Silica Fume (wt%)	Sand-to-Binder Ratio	Water-to-Binder Ratio	Steel Fiber Content (vol%)	PCE-SP (wt%)
20	1:1	0.20	2.5	2.5

**Table 3:** Pore size distribution of the specimens cured in different conditions.

Specimen	Pore Size Distribution (%)			
	<20 nm	20~100 nm	100~1000 nm	>1000 nm
RT-cured	21.8	27.7	13.1	37.4
−20 °C GJH-cured	15.5	32.8	19.7	32.0
−40 °C GJH-cured	16.2	24.2	22.8	36.8
−60 °C GJH-cured	15.7	12.5	11.1	60.7

## Data Availability

The original contributions presented in the study are included in the article; further inquiries can be directed to the corresponding author.
